# Unilateral Surgery for Medullary Thyroid Carcinoma: Seeking for Clinical Practice Guidelines

**DOI:** 10.3389/fendo.2022.875875

**Published:** 2022-07-11

**Authors:** Daqi Zhang, Carla Colombo, Hui Sun, Hoon Yub Kim, Antonella Pino, Simone De Leo, Giacomo Gazzano, Luca Persani, Gianlorenzo Dionigi, Laura Fugazzola

**Affiliations:** ^1^ Division of Thyroid Surgery, China-Japan Union Hospital of Jilin University, Jilin Provincial Key Laboratory of Surgical Translational Medicine, Jilin Provincial Engineering Laboratory of Thyroid Disease Prevention and Control, Changchun City, China; ^2^ Division of Endocrine and Metabolic Diseases, Istituto di Ricovero e Cura a Carattere Scientifico (IRCCS) Istituto Auxologico Italiano, Milan, Italy; ^3^ Department of Pathophysiology and Transplantation, University of Milan, Milan, Italy; ^4^ Korea University College of Medicine (KUMC) Thyroid Center, Department of Surgery, Korea University Hospital, Korea University College of Medicine, Seoul, South Korea; ^5^ Division of Surgery, Istituto Auxologico Italiano INstituto di Ricovero e Cura a Carattere Scientifico (IRCCS), Milan, Italy; ^6^ Department of Human Pathology of Adulthood and Childhood, University of Messina, Messina, Italy; ^7^ Pathology Unit, Istituto Auxologico Italiano INstituto di Ricovero e Cura a Carattere Scientifico (IRCCS), Milan, Italy; ^8^ Department of Medical Biotechnologies and Translational Medicine, University of Milan, Milan, Italy

**Keywords:** medullary thyroid cancer (MTC), lobectomy, calcitonin, surgery, thyroid cancer

## Abstract

Optimized preoperative diagnostic tools with calcitonin tests, ultrasound features, functional imaging modalities, and genetic testing to detect hereditary forms have led to an increased rate of earlier diagnosis and surgery for medullary thyroid cancer (MTC). This helps to adapt the primary surgery to the tumor stage and avoid surgical overtreatment for localized tumor growth, i.e., deviating from the regularly recommended thyroidectomy with bilateral central lymph node dissection in favor of a limited unilateral approach. To limit primary surgical therapy, it is crucial that the MTC is clinically unifocal, sporadic, and confined to the thyroid, and that calcitonin levels indicate biochemical recovery after surgery. The main requirement for such a limited approach is the availability of frozen section studies that reliably indicate (i) R0 resection of the MTC, (ii) absence of infiltration of the organ capsule, (iii) lack of desmoplasia (i.e., evidence of the metastatic potential of the MTC), (iiii) absence of contralateral disease or precancerous lesions. Informed consent is mandatory from the patient, who has been fully informed of the advantages, disadvantages, and potential risks of not undergoing the “classic” surgical procedure. The aim of this article is to review the guidelines for the management of early-stage MTC.

## Introduction

Medullary thyroid carcinoma (MTC) arises from the parafollicular cells or C cells of thyroid and accounts for 3%-5% of thyroid cancers (1). C cells migrate into the thyroid from the neural crest during embryonic life, reside in the basal layer of thyroid follicles, and account for 1% of thyroid cells. C cells are distributed throughout the thyroid gland, but are more concentrated in the area between the upper third and lower two-thirds of the lobes. They differ from the cells of the follicular epithelium primarily in their ability to secrete calcitonin (Ctn), a small single-chain peptide of 32 amino acids whose gene is located on the short arm of chromosome 11 (1, 2). The normal serum concentration of Ctn is less than 10 pg/mL, though different levels should be considered depending on the assays used. In the last 10 years, due to the unavailability of pentagastrin, calcium stimulation test is used in clinical practice to stimulate secretion of Ctn and to differentiate between neoplastic and non-neoplastic hypercalcitoninaemia. Occasionally, patients with C-cell hyperplasia or medullary microcarcinoma have only mildly elevated baseline levels Ctn, but these may markedly increase after stimulation with calcium (1, 2).

MTC occurs in most cases in a sporadic (80%) or hereditary (20%) form. Patients with sporadic MTC usually present with a thyroid nodule, which is associated with lymph node metastases in the neck region in about half of the cases. Distant metastases are rarely present at the time of diagnosis, and in these cases the organs most affected are usually the liver, lungs, and bones (3).

All patients with clinically manifest MTC have elevated baseline concentrations of Ctn.

The measurement of Ctn, recommended by some guidelines as a routine evaluation in the presence of thyroid nodules, has greater sensitivity for MTC diagnosis than cytology, which is not always conclusive.

Approximately 4-5% of cases of sporadic medullary carcinoma are actually hereditary. It is therefore necessary to perform genetic screening even in apparently sporadic cases to identify hereditary forms that have been misdiagnosed as sporadic (2). The hereditary form of MTC is inherited in an autosomal dominant manner and is due to a germline mutation of the proto-oncogene Rearranged during transfection (RET), which encodes a tyrosine kinase receptor. MTC can be the only clinical manifestation (familial MTC-FMTC) or can be combined to other diseases in the setting of a multiple endocrine syndrome (MEN 2). MEN 2A is a syndrome in which medullary carcinoma, pheochromocytoma (10-60%) and hyperparathyroidism (10-25%) are associated. Medullary carcinoma rarely occurs before 10 years of age in these patients and its prevalence increases with age. MEN 2B is a syndrome characterized by the association of medullary carcinoma, pheochromocytoma, ganglioneuromatosis (including mucinous neurinomas occurring in the distal part of the tongue, in the mucosa of the lips and throughout the gastrointestinal tract and possibly in the urinary tract), and marfanoid habitus (long and thin limbs, laxity of the ligaments and disproportionately long, thin limbs).

Genetic screening allows identification of family members of an affected individual who are also carriers of the mutated gene but are unaware of their disease and are destined to develop medullary thyroid carcinoma. In these individuals, it is therefore possible to intervene early with prophylactic or early thyroid surgery, which allows the disease to be prevented or cured if it has already manifested in a subclinical form. RET mutations screening should be performed in all first-degree family members of the proband. The analysis performed with Sanger sequencing or with Next Generation Sequencing (NGS) should include RET exons 5, 8, 10, 11, 13-16.

## Calcitonin Determination and Pre-Operative Staging

Evidence of MTC comes from a preoperatively elevated basal Ctn (bCtn). The grey area between a nonspecific or non-MTC-associated elevation of Ctn and an MTC can be narrowed down with modern assays: in women at 20-30 pg/ml and in men at 60-80 pg/ml (3–5). The Ctn stimulation test with calcium injection is recommended only in “borderline” cases (1, 6). Systematic recording of preoperative bCtn can comprehensively identify early stages of sporadic MTC (7). Ctn cut-off values indicating the presence of central or lateral LNM are hardly applicable to individual cases. Bae *et al.* report a bCtn of 266.6 pg/ml for ipsilateral central LNM with primary tumor < 1 cm, a bCtn of 755 pg/ml for contralateral central LNM, and 237.0 pg/ml for ipsilateral lateral LNM as cross-gender preoperative cut-off values (8). This information makes it clear that further criteria are required for the selection of the individually appropriate resection extent, which can be used to guide the surgical strategy.

Serum carcinoembryonic antigen (CEA) is usually elevated in advanced cases with distant metastases and, in addition, elevated levels of serum carbohydrate antigen 19.9 can be elevated in patients with MTC with a worse prognosis.

Patients with both sporadic and hereditary medullary carcinoma before surgery must be evaluated for the presence of pheochromocytoma by 24-hour urinary metanephrines and normetanephrines evaluation, and if they are elevated, it is appropriate to submit patient to abdominal CT or MRI (1, 2). The presence of primary hyperparathyroidism must also be ruled out by measuring calcium and parathyroid hormone levels. Moreover, neck ultrasound should be performed in all patients with MTC and further scans (CT, MRI or PET) are recommended only in selected cases (in patients with extensive neck disease and signs or symptoms of regional or distant metastases, and in all patients with a serum Ctn level greater than 500 pg/ml) (1).

## Surgery

Surgery is the only curative therapy for MTC. In contrast to thyroid carcinomas derived from the follicular epithelium, effective treatments such as radioiodine administration are not applicable. For this reason, total thyroidectomy with lymph node dissection is predominantly recommended as primary therapy (1, 2). While sporadic MTC are mostly unifocal tumors (80%), hereditary MTC (multiple endocrine neoplasms: MEN 2A, MEN 2B; familial MTC –FMTC-) are often multifocal and bilateral (9, 10).

Improvements in preoperative diagnostics, particularly the widespread preoperative determination of the sensitive and specific tumor marker as calcitonin (Ctn), have led to MTC being diagnosed and operated on earlier and thus at more favorable stages. De facto, Machens et al. showed in a 5-year comparison of primary tumor size of MTC how the proportion of tumors ≤ 1 cm increased from 19% to 39%, the incidence of lymph node metastases (LNM) decreased from 73% to 49%, and at the same time the biochemical cure rate increased from 28% to 62% (7).

The goal of primary surgery is “biochemical cure,” i.e., postoperative undetectable Ctn levels. This goal can be achieved in almost 100% of tumors confined to the thyroid gland without LNM. Once LNM and extrathyroidal growth (20%) are present, the chances of success decrease significantly (11). Machens *et al.* showed that in MTC, the number of LNM with systematic compartmental lymph node dissection is highly predictive of biochemical remission after surgery (12). In light of this, the recommendation for primary extensive lymph node dissection as a potential surgical overtreatment is increasingly criticized in the risk-benefit analysis. Here, the safer biochemical cure provided by radical initial surgery competes with the higher risk of hypoparathyroidism and recurrent laryngeal nerve palsy (13).

Recently, Miyauchi et al. to develop a diagnostic algorithm using simple hemithyroidectomy as a limited resection approach after ruling out hereditary MTC (9). This prospective study showed no bilateral multifocal tumor growth in 134 patients with sporadic MTC. Notably, all 65 patients who received hemithyroidectomy alone showed no MTC recurrence in the preserved thyroid lobe (14).

Raffel *et al.* demonstrated that limited resection may be sufficient for sporadic pT1 MTC in 15 patients with MTC diagnosed only postoperatively who had originally undergone subtotal resection mainly for nodular goiter (15). They pointed out the high complication rates of completion surgery leading to transient and permanent hypoparathyroidism (5.6%; 3.5-8.8%), vocal cord paralysis (3.8-8.5%; 2.8-8.4%) and transient Horner syndrome (3.8-5.6%) and recommended no radical surgery in their algorithm after an intraoperative frozen section examination for T1 MTC, but thyroidectomy with bilateral central and possibly unilateral or bilateral lateral connect lymph node dissection for MTC > 1 cm. Interestingly, a recent study based on the Korean NHIS database (16), showed that 34.6% of patients were submitted to lobectomy and the proportion did not change during the 2004 to 2016 period.

## Guidelines for Extent of Thyroid Resection

**Figure 1:**
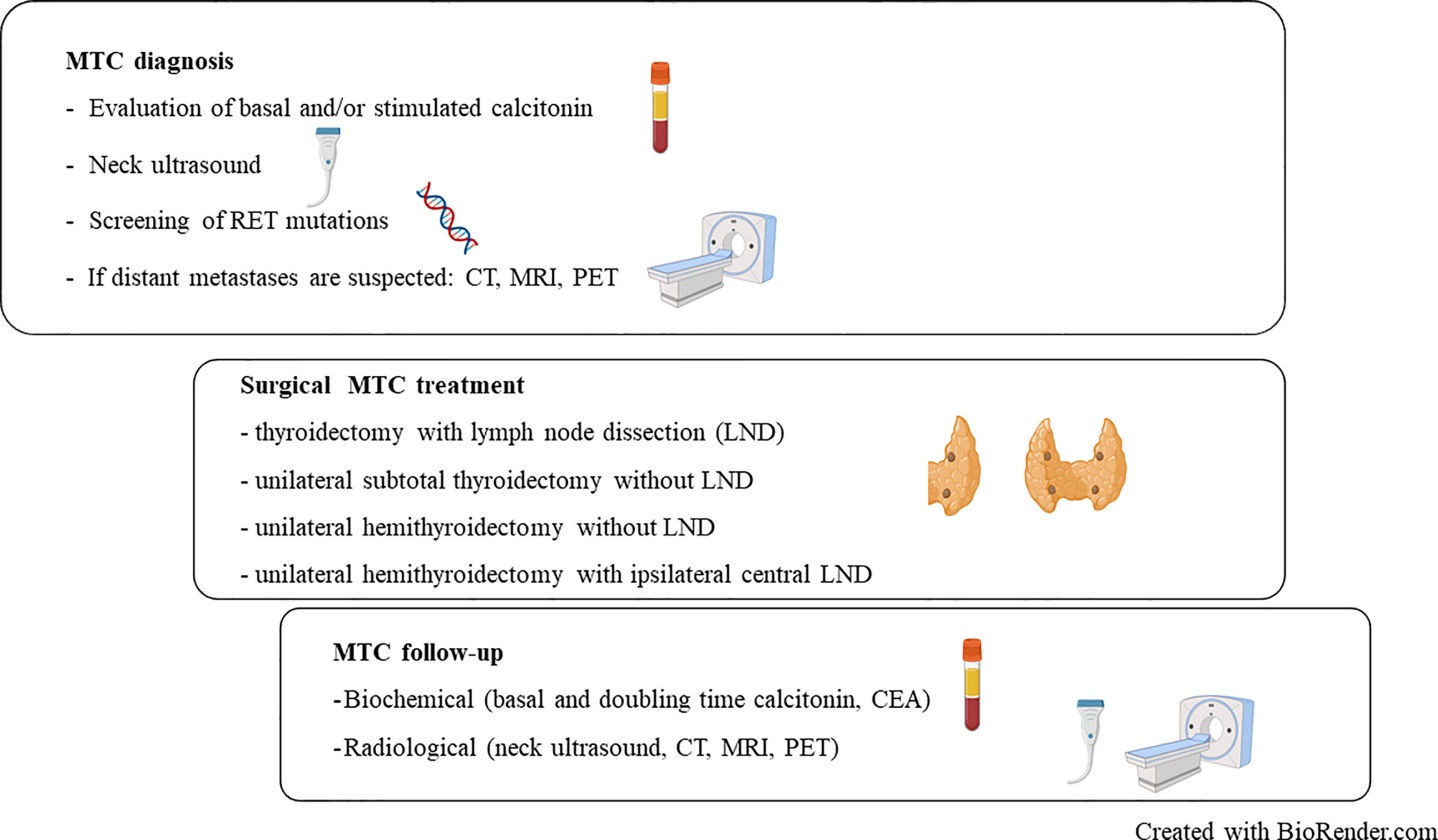
Summary overview of diagnosis, treatment and follow-up procedure in medullary thyroid carcinoma (MTC). RET, REarranged during Transfection; CT, Computerized Tomography; MRI, Magnetic Resonance Imaging; PET, Positron Emission Tomography; CEA, Carcinoembryonic antigen; LND, Lymph Node Dissection.

MTC can be confirmed by fine-needle aspiration cytology or diagnosed on the basis of a markedly elevated basal and/or stimulated Ctn, and suspicious ultrasound findings (**Figure 1**). If history and/or the genetic analysis excludes a hereditary MTC, the tumor has a high probability to be unifocal, even though in sporadic MTC multifocal tumor foci and/or bilateral tumor foci or areas of diffuse C-cell hyperplasia are found in up to 15% of cases. This is another reason why international guidelines consistently recommend thyroidectomy with central lymph node dissection as primary therapy for confirmed MTC (**Table 1**) (17–20).

**Table 1:** Recommendations of the major international guidelines concerning the type of surgery for the initial treatment of MTC.

Country (Society)	China (Expert Chinese consensus)	Korea (KATES)	USA (ATA)	England (BAETS)	Germany (CAEK)	USA (NCCN)
**Year**	2020	2017	2015	2016	2012	2014
**Reference**	35	36	1	37	2	38
**Preoperative Ctn determination**	Recommended for suspected pr-surgery MTC (Strong recommendation, Low-quality evidence)	According to presurgery evaluation (FNA consistent with MTC or familial MTC/MEN2, etc), not recommended routinely	According to medical assessment, not recommended routinely	Only when there is suspicion, not recommended routinely	Recommended before all TSs (“preoperative MTC screening ")	Recommended only for pre-surgery suspicion of MTC
**Type of thyroid surgery**	TT	TT	TT	TT	TT	TT
**Ipsilateral central compartment** **lymphadenectomy**	Routine (Level VI) (Strong recommendation, Low-quality evidence)	Routine (Level VI)	Routine (Level VI)	Routine (Level VI)	For clinical/imaging/FNA indication: attack-oriented bCtn 20–200 pg / ml: also clinically negative risk-oriented: routine (Level VI + VII)	Tumor <1cm + unilateral: "consider" Tumor ≥1cm or bilateral: routine (Level VI)
**Contralateral central compartment** **lymphadenectomy**	Routine (Level VI) (Strong recommendation, Low-quality evidence)	Routine (Level VI)	Routine (Level VI)	Routine (Level VI)	For clinical/imaging/FNA indication attack-oriented bCtn 20–200 pg / ml: also clinically negative; risk-oriented: routine (Level VI + VII)	- tumor <1cm + unilateral: - "consider" tumor ≥1cm or bilateral: routine (Level VI)
**Ipsilateral** **laterocervical lymphadenectomy**	-Prophylactic: depending on Tumor size and Ctn or at IOFS tumor positive central node (Week recommendation, Low-quality evidence) -If clinically suspected: Routine (Strong recommendation, Low-quality evidence)	-If clinically suspected in high risk patients or proven malignancy : routine -Prophylactic: no recommended routinely	-Prophylactic: dependent on Ctn (no Cut-off) -If clinically suspected: Routine	- Prophylactic: depending on Tumor size and Ctn or at IOFS tumor positive central node - T2-4: Routine selective prophylactic (Level IIa-Vb)	- bCtn 20–200 pg / ml: also clinically negative, - risk-oriented: routine (Level II-V)	Prophylactic: "consider" for high volume Center or extensive findings
**Contralateral laterocervical lymphadenectomy**	Not mentioned	Not mentioned	With tumor positive ipsilateral lateral dissection and if Ctn > 200 pg / ml	T2-4: Routine selective prophylactic (Level IIa-Vb)	bCtn> 200 pg / ml: risk-oriented recommended (Level II-V)	Not mentioned

MTC, medullary thyroid cancer; KATES, Korean Association of Thyroid and Endocrine Surgeons; ATA, American Thyroid Association; bCtn, basal Calcitonin, BAETS, British Association of Endocrine Surgeons; CAEK, Chirurgische Arbeitsgemeinschaft EndoKrinologie; Ctn, Calcitonin; FNA, fine needle aspiration; HT, hemithyroidectomy; IOFS, intraoperative frozen section; MTC, medullary thyroid carcinoma; NCCN, National Comprehensive Cancer Network; TS, Thyroid Surgery; TT, Total Thyroidectomy.

Registry data from Germany show that the recommendation for preoperative Ctn determination in nodular goiter was widely followed in 63% of cases (21). The higher preoperative detection rate of MTC as a result of Ctn screening improves the detection of those sporadic MTCs fated to become clinically evident tumors at an early stage, when extrathyroidal extension and/or local or distant metastases are less frequently present, thus improving prognosis (12). Surveillance, Epidemiology, and End Results (SEER) registry data also confirmed an increase up to 39% in micro-MTCs. Of the 310 micro-MTCs included (mean 5.7 mm), 31% had multifocal growth and 3.7% had growth outside the thyroid gland. In addition, LNM were present in 37% of patients and 5% had distant metastases (3). This highlights that tumor diameter or evidence of micro-MTC cannot be the sole criteria to base a limited resection strategy. Similarly, based on 766 patients with MTC confined to the central neck and treated with less than total thyroidectomy (TT), TT alone or TT with lymph node excision, Randle *et al.* found that the extent of initial resection may not significantly change disease specific survival (4). Therefore, it is controversial whether thyroidectomy and lymph node dissection is required in every case of MTC confined to the thyroid gland, especially for T1a MTC (9, 14).

The aim of the intraoperative histopathological frozen section examination is not only to confirm the diagnosis on the primary tumor in the case of a clinical suspicion, but also to assess crucial features such as size, location and extrathyroidal extension in order to select the best resection strategy (TT, lobectomy, associated or not with ipsilateral central lymph node dissection).

Two points are particularly relevant for the intraoperative histopathological assessment of the potential of an MTC for lymph node metastasis: (i) on the one hand, the extent of the intratumoral desmoplasia and (ii), on the other hand, the extent of the thyroid capsule invasion. Koperek *et al.* described desmoplasia as a reliably reproducible marker for the metastatic potential in MTC (22). The detection of desmoplastic stromal reaction correlated significantly with the presence of LNM, while all MTC without desmoplasia had no LNM. In addition, tumor diameter, tumor stage, angioinvasion, and tumor lymphocyte infiltration were associated with LNM. So far, these data have only been independently confirmed for large, sporadic MTCs with a primary tumor size of over 1 cm (23), while smaller tumors are more suitable for a limited surgical approach (24). Desmoplasia and the degree of thyroid capsule rupture indicate aggressive growth behavior with a corresponding potential for metastasis in MTC. If the thyroid capsule is not or only slightly reached or broken focally and desmoplasia is not or only slightly detectable, the risk of metastasis can be considered as low. The reliable assessment of both criteria on frozen sections is a prerequisite for limiting the surgical resection extent while preserving the oncological adequate result. It should be mentioned that the limited possibilities of the frozen section examination often do not give a clear result, especially in micro-MTCs. Unfortunately, other parameters correlated to proliferation activity (Ki-67 index, number of mitoses, necrosis, amyloid deposition, etc.) and prognostically associated with long-term survival rather than with disease persistence or structural recurrences (25), cannot be recorded in the frozen sections. Thus, it is worth to note that the intraoperative frozen section assessment should be used just as an additional factor to the gold standard pre-surgical assessments such as serum Ctn and CEA values and imaging results.

## Postoperative Follow-Up

If the guideline recommendations for routine Ctn screening were implemented, diagnosed MTCs should predominantly be microcarcinomas or pT1 stages. There is room for interpretation of the Ctn gray zone in preoperative Ctn determination in the absence of typical ultrasound or other imaging criteria. This means that limited resection may be sufficient even for MTC confirmed only postoperatively if:

- the tumor was R0-resected;
- the postoperative bCtn is lower than the preoperative;
- germline RET mutations analyses excludes hereditary MTC.

In principle, in the case of hereditary MTC and postoperative undetectable bCtn, a limited form of resection can be accepted, provided that regular sonographic, clinical, and laboratory follow-up is warranted. It should be noted that postoperative normalization of tumor marker Ctn takes time depending on the stage. Machens *et al.* showed that this occurred at least one week postoperatively in MTC with LNM and preoperative Ctn level below 500 pg/ml, at least 14 days postoperatively in MTC with LNM and preoperative Ctn between 500.1-1000 pg/ml, and at a later time point in MTC with > 10 LNM and MTC with LNM and preoperative Ctn > 1000 pg/ml (6).

In 105 Brazilian MTC patients, Ctn was undetectable after the first operation: the nadir of postoperative bCtn was 63% at 1 month and 98% at 6 months (26–30).

It is worth to mention that during post-surgery follow-up, measurement of CEA is not necessary in cases of undetectable levels of serum Ctn. On the contrary, serum CEA levels doubling time, similarly to those obtained for Ctn, is a useful predictors of survival and disease progression (31).

## Limited Forms of Resection for Intrathyroidal MTC

The trend toward more favorable primary MTC stages leads to the need to avoid overtreatment by adapting the resection strategy on a case-by-case basis. Prerequisites for this are the comprehensive clinical and laboratory diagnosis of the individual case as well as the structural availability of qualified pathologists, in order to obtain an intraoperative frozen section analysis though considering the above reported limitations (32–35). If the preoperative criteria indicate a sporadic, unifocal, and with expected biochemical cured MTC, and the intraoperative frozen section excludes desmoplasia and capsular infiltration or finds them only to a minor extent, the resection could be limited. Depending on other criteria (bCtn, location of the tumor in the thyroid lobe, clinical evidence of LNM, age of the patient), the following types of resection can be considered in deviation from the classical thyroidectomy concept with lymph node dissection (LND):

- unilateral subtotal thyroidectomy without LND;
- unilateral hemithyroidectomy without LND;
- unilateral hemithyroidectomy with ipsilateral central LND.

To date, apart from the Japanese prospective study by Miyauchi and Ito, there are few data that have examined the outcome of limited resection strategies for MTC. In particular, there is a lack of data on clinical series that have followed such an approach using intraoperative frozen sections and prospectively collected long-term outcomes on disease persistence or recurrence. In an interim analysis of the DGAV (German Society for General and Visceral Surgery) CASMED study - StudDoQ Thyroid/Parathyroid Registry **(** **Table 2** **)**, it was shown that limited resections were performed in 28 of 343 (8%) patients with MTC, including 75% hemithyroidectomies. T1 stages predominated, but multifocal tumors, T2 and T3 stages, as well as lymph nodal involvement and hereditary cases were also represented. Vocal cord paresis was found in 9 cases (32%) after surgery. Limited resection for palliative care was done in 2 cases. Intraoperative frozen section examination was performed only in 10 surgical specimens, in which MTC was detected in 4 cases, while 6 cases remained without evidence of malignancy. Oncologic completion surgery is reported for 10 (36%) cases at further follow-up, although whether this was due to the diagnosis of an MTC, TNM stage, or postoperative elevated Ctn remains open. Given the paucity of data, no general recommendation for a limited approach can be made at this time.

**Table 2:** Clinical, pathological and biochemical features of MTCs treated with different types of surgery in the interim analysis of the DGAV (German Society for General and Visceral Surgery) CASMED study - StudDoQ Thyroid/Parathyroid Registry.

Thyroid surgery type	n.	LND	Preoperative Ctn (pg/ml)	MTC TNM	RET screening	Postoperative Ctn (pg/ml)	Complications
Unilateral sTT	2	Unilateral CCND+LND (n= 1)	23; 66643	T1aN0 T1bN1	NS Negative	7; 84257	Ca/Vit D substitution None
Unilateral HT	21	-Selective diagnostic (n= 2) -Unilateral CCND (n= 5) -Unilateral CCND+LND (n= 1) -Bilateral CCND+LND (n= 1)	NS (n=6) 2;14;15;37 (n= 2);69;73;80; 110;258;276; 540;750;1900; 2150	T1aN0 (n= 8) T1aNx (n= 3) T1bNx (n=1) T1bN0 (n= 1) T1bmN1a (n= 1) T1bN1b (n= 1) T2N0 (n= 4) T3aN1b (n= 1) T3bmN1b (n= 1)	NS (n= 14) Negative (n= 4) MEN 2a (n= 3)	NS (n= 16) 1;3;5;8;18	NS (n=2) None (n= 11) VCP (n= 8)
HT + enucleation	3	NS	NS 61; 5	T1bN0; T1aNx; T1aN0	NS Negative NS	NS NS NS	Ca/Vit D substitution VCP
HT + sTT	2	Unilateral CCND (n= 1)	0; 763000	T1aNX; T2N1	NS NS	NS 5	NS None

Ctn, Calcitonin (normal value: <10 pg/ml); CCND, Central compartment node dissection; HT, Hemithyroidectomy; LND, lymph node dissection; MEN, Multiple Endocrine Neoplasia; MTC, Medullary Thyroid Carcinoma; n, Number; NS, Not Specified; VCP, Vocal Cord Palsy; sTT, subTotal Thyroidectomy.

Concerning the follow-up of patients undergoing hemithyroidectomy, available data are still scanty because the most commonly used surgical treatment to date has been TT. However, as for cancer arising from the follicular epithelium, these patients should be followed up by evaluating the trend of serum markers levels and by accurate neck ultrasound exams. Only in patients in whom Ctn and/or CEA are particularly high, with rising levels during follow-up, more in-depth imaging studies such as CT or PET scans must be performed **(** **Figure 1** **)**.

Therefore, it is suggested to document such limited operations and their long-term course promptly in prospective studies of the established registries. Nevertheless, based on the currently available data, a shared decision-making discussion in a multidisciplinary team should be made in individual cases in order to evaluate if a limited but oncological adequate resection can be proposed for a suspected MTC (35) **(** **Figure 1** **)**.

## Data Availability Statement

The raw data supporting the conclusions of this article will be made available by the authors, without undue reservation.

## Author Contributions

All authors listed have made a substantial, direct, and intellectual contribution to the work and approved it for publication.

## Funding

This study was supported by the EUROCRINE project, Grant No: 2022_01_25_05 Istituto Auxologico Italiano and by Ricerca Corrente Istituto Auxologico Italiano IRCCS (THY-CANC, 2022_03_08_03).

## Conflict of Interest

The authors declare that the research was conducted in the absence of any commercial or financial relationships that could be construed as a potential conflict of interest.

## Publisher’s Note

All claims expressed in this article are solely those of the authors and do not necessarily represent those of their affiliated organizations, or those of the publisher, the editors and the reviewers. Any product that may be evaluated in this article, or claim that may be made by its manufacturer, is not guaranteed or endorsed by the publisher.
